# The role of emergency medicine clerkship e-Portfolio to monitor the learning experience of students in different settings: a prospective cohort study

**DOI:** 10.1186/s12245-018-0184-9

**Published:** 2018-04-12

**Authors:** Arif Alper Cevik, Sami Shaban, Margret El Zubeir, Fikri M. Abu-Zidan

**Affiliations:** 1 Department of Internal Medicine, Emergency Medicine Clerkship, College of Medicine and Health Sciences, United Arab Emirates University, Al Ain, 17666 United Arab Emirates; 2 Department of Emergency Medicine, Tawam-John Hopkins Hospital, Al Ain, UAE; 3 Department of Medical Education, College of Medicine and Health Sciences, United Arab Emirates University, Al Ain, United Arab Emirates; 4 Department of Surgery, College of Medicine and Health Sciences, United Arab Emirates University, Al Ain, United Arab Emirates

**Keywords:** Emergency medicine, Undergraduate medical education, e-Portfolio, Electronic logbook

## Abstract

**Background:**

Although emergency departments provide acute care learning opportunities for medical students, student exposure to recommended curriculum presentations and procedures are limited. In this perspective, clinical environments providing learning opportunities for students should be monitored as part of an ongoing quality improvement process. This study aims to analyze student exposures and their involvement levels in two different hospitals (Tawam and Al Ain) so as to improve the teaching and learning activities.

**Methods:**

This is a prospective study on all 76 final year medical students’ electronic logbooks (e-Portfolio) of the academic year 2016/2017.

**Results:**

Students recorded 5087 chief complaints and 3721 procedures. The average patient and procedure exposure in a shift per student in Al Ain Hospital compared with Tawam Hospital were 7.2 vs 6.4 and 5.8 vs 4.3, respectively. The highest full involvement with presentations was seen in the pediatric unit (67.1%, *P* < 0.0001). Urgent care shifts demonstrated the highest area of “full involvement” with procedures for our students (73.2%, *P* < 0.0001). Students’ highest involvement with presentations and procedures were found during the night shifts (*P* < 0.0001, 66.5 and 75.1%, respectively).

**Conclusions:**

The electronic portfolio has proven to be a very useful tool in defining the learning activities of final year medical students during their emergency medicine clerkship and in comparing activities in two different clinical settings. Data collected and analyzed using this e-Portfolio has the potential to help medical educators and curriculum designers improve emergency medicine teaching and learning activities.

## Background

Emergency departments (EDs) provide rich clinical learning opportunities to medical students [1]. They increase confidence in both acute care and procedural skills [2]. Nevertheless, their exposure to recommended curriculum presentations and procedures are limited [3–6]. In addition, their involvement level with clinical presentations and procedures varies [7]. Alignment of curriculum content, learning outcomes, teaching and learning activities, and assessments is critically important in reaching desired outcomes. Given this perspective, clinical environments providing learning opportunities for students should be monitored to determine if they meet expectations and are fit for purpose. Exposures including quantity and severity differ between various settings. Without understanding student exposure to presentations and procedures in the clinical environment, we cannot assure that they receive proper training in the clerkships [5], neither can we modify the curriculum to fit their needs.

Clinical logbooks document medical student clinical exposures and improve student performance of procedures [8, 9]. Timely student learning exposure is very important during short clerkships because it can provide feedback to students and guide them to achieve their required presentations and procedures. Hard copy logbooks may not be useful for this purpose. Our previous experience showed that collecting, evaluating, and analyzing logbook data at the end of the clerkship are difficult and not useful for timely feedback [7]. Electronic logbooks overcome many of these obstacles [10]. Proper monitoring and analysis of electronic logbooks can modify teaching and learning activities during the clerkship as well as provide guidance for future modifications of the curriculum.

In this study, we aimed to prospectively analyze student ED exposures and involvement levels in two hospitals during various shifts and units in order to improve curriculum teaching and learning opportunities in our clerkship.

## Methods

### Study design, setting, and participants

This is a prospective study analyzing student electronic logbooks (e-Portfolio). The study was conducted in the Emergency Medicine (EM) clerkship of the College of Medicine and Health Sciences of the UAEU during 2016/2017 academic year. The EM clerkship is a 4-week rotation for senior (sixth year) medical students. The content of the EM clerkship curriculum was adopted from the Society for Academic Emergency Medicine [11] and International Federation for Emergency Medicine [12] and consists of 11 chief complaints (abdominal pain, altered mental status, cardiac arrest and arrhythmias, chest pain, fever in a child, gastrointestinal bleeding, headache, poisoning, respiratory distress, shock, and major trauma) and five procedures (airway management, cardiopulmonary resuscitation and arrhythmia management [CPR-AM], suturing and wound care, focused assessment with sonography on trauma [EFAST], rapid ultrasonography for shock and hypotension [RUSH]).

Teaching, learning, and assessment activities are directed by a clerkship director and implemented collaboratively with core-faculty members and senior EM residents. Teaching sessions include up to date pedagogies such as flipped classroom, team-based learning, and case-based learning instead of didactic faculty lectures. Skill sessions are held in the College simulation and skills center. Simple medical skills manikins are used for airway and suturing practices as well as high-fidelity manikins for CPR-AM practices. Normal human models are used for ultrasound training. Assessments consist of weekly multiple-choice question exams in team-based learning activities, oral case presentations, clinical shift performance evaluations by supervisors, a final multiple-choice question exam, and an objective-structured clinical examination.

The students complete their clinical shifts in two teaching hospitals in our city, Tawam Hospital and Al Ain Hospital. Both hospitals treat more than 115,000 emergency patients every year. Tawam Hospital implements an ACGME-I accredited EM residency program. During the 2016/2017 academic year, there were five groups of 9 to 17 students each, with a total of 76 final year medical students (47 females, 19 males). Six out of ten clinical shifts for each medical student were completed in Tawam Hospital, and each clinical shift period was 9 h long. The shift hours were also similar in the Al Ain Hospital. Students complete three clinical shifts in the resuscitation unit, four in the urgent care area, two in the fast-track area, and one in the pediatric unit. Students work different shift times in the units; day time (08:00–17:00), three shifts; prime time (13:00–22:00), four shifts; evening time (15:00–24:00), two shifts; and night time (24:00–09:00), one shift.

### Data collection and analysis

SharePoint was used as the e-Portfolio medium of EM clerkship. Students access this website securely from their computers or mobile devices by entering their specific credentials [13]. Students are asked to complete a minimum of 33 chief complaints and 33 procedures during the 4-week period. At the beginning of the clerkship, the clerkship director oriented the students on how to fill in the e-Portfolio. Students were informed to enter the main/chief complaint of the patients and most critical/life or organ-saving procedures first into the e-Portfolio. They were free to log all chief complaints and procedures they encounter on patients.

Data in the logbooks included descriptive information of patients such as matriculation number, age and gender, chief complaint seen, and procedure exposed by the student, hospital, unit, shift time, student involvement level, and some specific clinical areas related to their patients. Student involvement had three levels: (1) observation alone (observation with minimal activity), (2) partial involvement (first assistant with up to 50% activity), and (3) full involvement (start to finish care with more than 50% activity).

Students were supervised by an attending physician or a senior resident during the clinical shifts. Students were informed to seek help from the supervisors when deciding on data entry of any of the fields, especially the involvement level. Supervisors were the final authority to accept, modify, or cancel the logbook entries according to their own judgment. Supervisors also evaluated each student for their clinical performance at the end of clinical shifts.

This online platform provides continuous monitoring of student logbook entries by the clerkship director and offers feedback opportunities to students. The clerkship director used the log results in the individual feedback sessions which were held in the second week of the clerkship. Results of the e-Portfolio were evaluated at the end of the clerkship.

Comparison of continuous data of two groups was performed using the Mann–Whitney *U* test. Comparison of categorical data was performed using Pearson chi-squared test or Fisher’s exact test as appropriate. A *P* value of less than 0.05 was accepted as significant. Data were analyzed using Statistical Package for the Social Sciences (IBM-SPSS version 24.0, Chicago, IL, USA).

## Results

Seventy-six students recorded 5087 chief complaints with more patients seen in Tawam Hospital, 2903 (57.1%). There were slightly more male patients logged by students (52.1%). Table 1 shows the comparison of patient demographics, chief complaints, and student involvement between the two hospitals. The total number of procedure encounters logged by students was 3721 with more procedures performed in Tawam Hospital 1971 (52.9%). Procedures were performed more often on male patients (55.8%). Table 2 shows the comparison of patient demographics, procedures, and student involvement between the two hospitals.

**Table 1:** Patients, chief complaints, and student involvement in the two hospitals

Categories	Tawam Hospital *N* = 2903 (%)	Al Ain Hospital *N* = 2184 (%)	Total *N* (%)	*P* value
Patient gender				< 0.0001
Male	1416 (48.8)	1234 (56.5)	2650 (52.1)	
Female	1487 (51.2)	950 (43.5)	2437 (47.9)	
Patient age	25.0 (0.08–112)	36.0 (0.08–106)		< 0.0001
Patient age category				< 0.0001
0–17	1159 (39.9)	228 (10.4)		
18–65	1304 (44.9)	1673 (76.6)		
> 65	440 (15.2)	283 (13.0)		
Chief complaints *n* (%)				< 0.0001
Abdominal pain	476 (16.4)	562 (25.7)	1038 (20.4)	
Chest pain	199 (6.9)	261 (12.0)	460 (9.0)	
Respiratory distress	234 (8.1)	118 (5.4)	352 (6.9)	
Fever in child	271 (9.3)	1 (0.0)	272 (5.3)	
Headache	129 (4.4)	107 (4.9)	236 (4.6)	
Major trauma	80 (2.8)	147 (6.7)	227 (4.5)	
Altered mental status	75 (2.6)	63 (2.9)	138 (2.7)	
Poisoning	20 (0.7)	22 (1.0)	42 (0.8)	
Shock	25 (0.9)	16 (0.7)	41 (0.8)	
Gastrointestinal bleeding	19 (0.7)	17 (0.8)	36 (0.7)	
Cardiac arrest	18 (0.6)	11 (0.5)	29 (0.6)	
Others	1357 (46.7)	859 (39.3)	2216 (43.6)	
Student involvement *n* (%)				< 0.0001
Observation	321 (11.1)	167 (7.6)	488 (9.6)	
Partial	793 (27.3)	638 (29.2)	1431 (28.1)	
Full	1789 (61.6)	1531 (63.1)	3168 (62.3)	
Shift time				< 0.0001
Day	594 (20.5)	500 (22.9)	1094 (21.5)	
Prime	1068 (38.8)	862 (39.5)	1930 (37.9)	
Evening	1137 (39.2)	279 (12.8)	1416 (27.8)	
Night	104 (3.6)	543 (24.9)	647 (12.7)	
Location				< 0.0001
Resuscitation	633 (21.8)	481 (22.0)	1114 (21.9)	
Fast track	795 (27.4)	26 (1.2)	821 (16.1)	
Urgent care	849 (29.2)	1667 (76.8)	2526 (49.7)	
Pediatric	626 (21.6)	0 (0.0)	626 (12.3)	

Data are presented as median (range) for patient age and number (%). *P* value using Mann–Whitney *U* test, Pearson’s chi-square, or Fisher’s exact test as appropriate

**Table 2:** Patients, procedures, and student involvement in the two hospitals

Categories	Tawam Hospital *N* = 1972 (%)	Al Ain Hospital *N* = 1749 (%)	Total *N* (%)	*P* value
Patient gender				< 0.0001
Male	1030 (52.2)	1048 (59.9)	2078 (55.8)	
Female	942 (47.8)	701 (40.1)	1643 (44.2)	
Patient age	30.0 (0.08–107)	36.0 (0.00–107)		< 0.0001
Patient age category				< 0.0001
0 – 17	642 (32.6)	165 (9.4)		
18 – 65	929 (47.1)	1348 (77.1)		
> 65	401 (20.3)	236 (13.5)		
Procedures				< 0.0001
Suturing lacerations	147 (7.5)	116 (6.6)	263 (7.1)	
Wound care*	164 (8.5)	96 (5.4)	260 (7.0)	
EFAST	56 (2.8)	83 (4.7)	139 (3.7)	
Airway management	80 (4.1)	29 (1.7)	109 (2.9)	
RUSH	51 (2.6)	21 (1.2)	72 (1.9)	
CPR	15 (0.8)	11 (0.6)	26 (0.7)	
Defibrillation/cardioversion	5 (0.3)	4 (0.2)	9 (0.2)	
Other	1454 (73.7)	1389 (79.4)	2843 (76.4)	
Student involvement				< 0.0001
Observation	336 (17.0)	177 (10.1)	513 (13.8)	
Partial	331 (16.8)	279 (16.0)	610 (16.4)	
Full	1305 (66.2)	1293 (73.9)	2598 (69.8)	
Shift time				< 0.0001
Day	506 (25.7)	384 (22.0)	890 (23.9)	
Prime	730 (37.0)	710 (40.6)	1440 (38.7)	
Evening	659 (33.4)	209 (11.9)	868 (23.3)	
Night	77 (3.9)	446 (25.5)	523 (14.1)	
Location				< 0.0001
Resuscitation	661 (33.5)	474 (27.1)	1135 (30.5)	
Fast track	549 (27.8)	28 (1.6)	577 (15.5)	
Urgent care	534 (27.1)	1247 (71.3)	1781 (47.9)	
Pediatric	228 (11.6)	0 (0.0)	228 (6.)	

Data are presented as median (range) for patient age and number (%). *P* value using Mann–Whitney *U* test, Pearson’s chi-square, or Fisher’s exact test as appropriate

*Wound care consists of burn care, simple dressing, steri-strip, and glue application

**Fig. 1:**
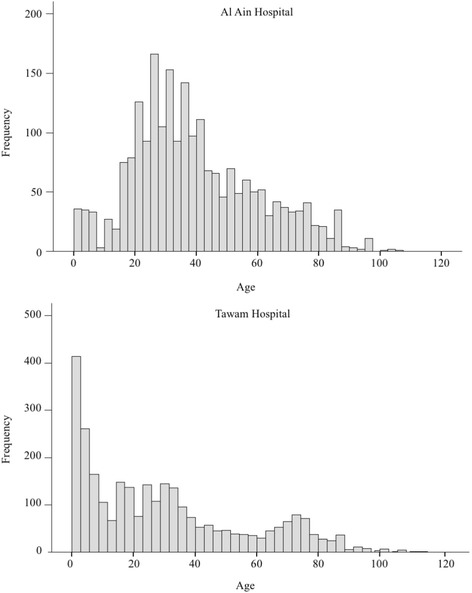
Histogram of patients’ age who were encountered by the sixth year medical students in the emergency medicine clerkship in Al Ain (**a**) and Tawam Hospitals (**b**)

Students were exposed to younger patients in Tawam Hospital (Fig. 1). The distribution of chief complaints, procedures, and student involvement levels were significantly different between the two hospitals (*P* < 0.0001). There were 2216 (43.6%) encountered chief complaints in the “other” category which was not included in recommended curriculum topics (Appendix 1). Our students logged 2843 (76.4%) of procedures in the category of “other” (Appendix 2). Student involvement with chief complaints and procedures were significantly different between units (Table 3, *P* < 0.0001). The highest full involvement with presentations was seen in the pediatric unit (67.1%, *P* < 0.0001). Urgent care shifts demonstrated the highest area of “full involvement” with procedures for our students (73.2%, *P* < 0.0001). Student involvement with chief complaints and procedures were significantly different between shift times (Table 4, *P* < 0.0001). Students’ highest involvement with presentations and procedures were found during the night shifts (*P* < 0.0001, 66.5 and 75.1%, respectively).

**Table 3:** Students’ involvement in different emergency department units

Categories	Resuscitation *N* (%)	Fast track *N* (%)	Urgent care *N* (%)	Pediatric *N* (%)	*P* value*
Chief complaint					< 0.0001
Observation	114 (10.2)	107 (10.3)	223 (8.8)	44 (7.0)	
Partial	336 (32.9)	196 (23.9)	707 (28.0)	162 (25.9)	
Full	634 (56.9)	518 (63.1)	1596 (63.2)	420 (67.1)	
Total	1114 (100)	821 (100)	2526 (100)	626 (100)	
Procedure					< 0.0001
Observation	159 (14.0)	106 (18.4)	207 (11.6)	41 (18.0)	
Partial	222 (19.6)	73 (12.7)	271 (15.2)	44 (19.3)	
Full	754 (66.4)	398 (69.0)	1303 (73.2)	142 (62.7)	
Total	1135 (100)	577 (100)	1781 (110)	228 (100)	

*This is the overall *P* value of Pearson’s chi-square for the 3 × 4 tables of the level of involvement in the chief complaints and procedures for different units

**Table 4:** Shift times and student involvement in emergency medicine clerkship

Categories	Day *N* (%)	Prime *N* (%)	Evening *N* (%)	Night *N* (%)	*P* value*
Chief complaint					< 0.0001
Observation	117 (10.7)	202 (10.5)	121 (8.5)	48 (7.4)	
Partial	330 (30.2)	521 (27.0)	411 (29.0)	169 (26.1)	
Full	647 (59.1)	1207 (62.5)	884 (62.4)	430 (66.5)	
Total	1094 (100)	1930 (100)	1416 (100)	647 (100)	
Procedure					< 0.0001
Observation	148 (16.6)	188 (13.1)	131 (15.1)	48 (8.8)	
Partial	154 (17.3)	223 (15.5)	149 (17.2)	84 (16.1)	
Full	588 (66.1)	1029 (71.5)	588 (67.7)	393 (75.1)	
Total	890 (100)	1440 (100)	868 (100)	523 (100)	

*This is the overall *P* value of Pearson’s chi-square for the 3 × 4 tables of the level of involvement in the Chief complaints and procedures for different units

## Discussion

Our study has shown that on average, the number of chief complaints and procedures exceeded the minimum required in our curriculum. However, e-Portfolio results showed that our students did not encounter all the recommended presentations and procedures of the curriculum in the clinical environment. The two hospitals showed differences in patient gender, age, chief complaint, procedure distribution, student involvement, and encounters related to shift time and location. Student involvement also varied depending on the location and clinical shift time.

We can enhance the learning experience of students in our curriculum in two ways. First, we can increase the feedback frequency to students regarding their logbook entries and guide them during the clerkship on recommended missing topics and procedures. In fact, this is what we did, individual feedback sessions included details of student logs at the midpoint of the course. Students were reminded about recommended presentations and procedures of the curriculum. In our recently published study, students who used hard copy logbooks encountered 57.8% of presentations in the “other” category [7]. This ratio decreased to 46.7% in the current study. It is possible that e-Portfolio record availability for students and individual feedback sessions using these records helped the students to focus on the recommended topics compared with students who used hard copies and did not have this feedback opportunity during the clerkship.

Second, we can modify teaching and learning activities by timely and focused feedback based on first-hand data and remediable issues will be beneficial for students [14]. Our results gave us a chance to modify our individual feedback sessions on a weekly basis. Students were exposed to a 1-hour case discussion session for each chief complaint and 90-min practical skills session for each procedure in the teaching program. However, they were exposed differently in the hospitals to these topics. As an example, our least encountered topics are cardiac arrest, gastrointestinal bleeding, shock, poisoning, and altered mental status in the chief complaint category and defibrillation/cardioversion, CPR, RUSH protocol, and airway management in the procedure category. It is obvious that the less exposed topics should be reviewed more in the classroom or trained on in the simulation/skills practices. In the classroom, interactive case discussions may connect knowledge with the clinical environment, encourage self-regulated learning, and identifies the gaps in knowledge or skills [15–17]. Simulation activities improve students’ knowledge, performance, confidence, and satisfaction [18–20]. Chakravarthy et al.’s systematic review concluded that simulation activities in clerkships are useful although future studies are needed to determine their efficacy [21].

Modifying shift times and locations can also be an option to improve teaching and learning. As an example, we reviewed the chest pain topic on the first day of the clerkship. This complaint was encountered more in Al Ain Hospital (12.0 vs 6.9%) although students have less shifts in this hospital. As a modification, students were given more shifts in this hospital in the first week of the clerkship. Al Ain Hospital has no EM residency program, and the ED is considered a community-based hospital ED. The average patients and procedure in a shift at Al Ain Hospital were more than those at Tawam Hospital. These results supported DeLahunta’s study in which they found that students assess more patients and perform procedures significantly more at a community-based hospital [22]. Furthermore, changing the distribution of shifts from one hospital to another will not affect the final exam results. Bernard reported that clerkship clinical site does not affect final exam score and using community hospitals for clinical teaching does not compromise education [23]. A similar approach can be applied to shift time. It is known that night shifts offer students extra educational opportunities [24]. Our students show significant full involvement during the night shifts. Therefore, increasing night shifts can improve student engagement with patients. However, modifying clinical schedules to improve performance requires higher and multi-level analysis and programming [25].

### Limitations

We have to acknowledge that our study has several limitations. Although our clinical supervisors do their best to assure the validity of our data, the control of every entry in busy clinical shifts cannot be completely assured. In addition, underreporting of core problems by students as described by Denton is also one of our continuous concerns [7, 26]. The present study includes a single EM clerkship in a medical college and covers only a 1-year period. Thus, the results may not be generalizable to different settings.

As adult learners, students decide their own learning needs and choose presentations and procedures relevant to these needs. However, achieving sufficient exposure to the recommended curriculum topics is important to achieve the intended learning outcomes. Students may not reach educationally acceptable levels of presentations and procedures during their clerkship. Therefore, some modifications in clerkship curricula may turn out to be necessary.

Because there is no defined number of exposures and level of involvement to achieve student competency on a specific chief complaint or procedure in the literature [7], our changes in teaching and learning applications in the curriculum depend on our own assumptions. Using multi-level analyses and programming will provide a better understanding of the effects of modifying the ED student scheduling. We are aiming in the near future to make changes that achieve the best desired clinical experience for our students.

## Conclusions

The electronic portfolio has proven to be a very useful tool in defining the learning activities of final year medical students during their emergency medicine clerkship and in comparing activities in two different clinical settings. Data collected and analyzed using this e-Portfolio has the potential to help medical educators and curriculum designers improve EM teaching and learning activities.

## Appendix 1

**Table 5:** “Other” chief complaints encountered during the emergency medicine clerkship

Chief complaints	*N* (%)
Minor trauma	927 (41.8)
Infectious problems	685 (30.9)
Neurologic problems	283 (12.8)
Orthopedic problems	228 (10.3)
Uncategorized	22 (1.0)
Gastrointestinal problems	19 (0.9)
Obstetric/gynecologic/urogenital	19 (0.9)
Head and neck problems	14 (0.6)
Cardiovascular problems	6 (0.3)
Respiratory problems	5 (0.2)
Skin and lymph problems	4 (0.2)
Allergic problems	3 (0.1)
Endocrine problems	1 (0.0)
Total	2216 (100)

## Appendix 2

**Table 6:** “Other” procedures encountered during the emergency medicine clerkship

Procedure	*N* (%)
IV line placement	1536 (54.0)
ECG recording/reading	661 (23.3)
Blood drawing and ABG	194 (6.8)
Splints sprains/fractures	137 (4.8)
Urinary catheter placement	82 (2.9)
Reductions of dislocations	58 (2.0)
Incision/drainage of abscess	46 (1.6)
Sedation/analgesia	41 (1.4)
Naso/orogastric placement	24 (0.8)
Lumbar puncture	20 (0.7)
Other procedures	15 (0.5)
Other, ultrasound	14 (0.5
Fast strep test	10 (0.4)
Foreign body removal	5 (0.5)
Total	2843 (100)

## Abbreviations

ACGME-I: Accreditation Council of Graduate Medical Education-International
CPR-AM: Cardiopulmonary resuscitation–arrhythmia management
ED: Emergency department
EFAST: Extended focused assessment with ultrasound in trauma
EM: Emergency Medicine
RUSH: Rapid ultrasound in shock and hypotension
UAEU: United Arab Emirates University
